# The unsung hero: *ntnh* gene as complementary botulism marker

**DOI:** 10.3389/fcimb.2026.1758429

**Published:** 2026-02-23

**Authors:** Sylvia Valdezate, Mónica Valiente, Sergio Díaz-Ramón, Gema Carrasco, María J. Medina-Pascual, Javier Pardos, María Isabel Hurtado, Noelia Garrido, Pilar Villalón, Antonio J. Martín-Galiano

**Affiliations:** 1 Reference and Research Laboratory for Taxonomy, National Centre of Microbiology, Instituto de Salud Carlos III, Majadahonda, Madrid, Spain; 2 Veterinary Unit, Animal Department, Instituto de Salud Carlos III, Majadahonda, Madrid, Spain; 3 Proteomics Unit, Core Scientific and Technical Units, Instituto de Salud Carlos III, Majadahonda, Madrid, Spain

**Keywords:** BoNT, botulism, foodborne botulism, infant botulism, neurotoxin, Ntnh

## Abstract

Botulism is a rare but severe neurological disease caused by botulinum neurotoxins (BoNTs). Standard diagnostic methods including the mouse bioassay (SMB) and *bont* gene type-specific PCR, are often limited by the high genetic diversity among *bont* subtypes, which can lead to false-negative results. The nontoxin-nonhemagglutinin (*ntnh)* gene is highly conserved and exclusively co-located with the *bont* gene complex. Thus, this study evaluates the use of *ntnh* gene as a complementary diagnostic tool for botulism and assesses its association with BoNT types. The *ntnh* gene was detected in a prospective BoNT-diagnostic group (n=88) and a BoNT-historical group (n=54). Toxin cluster proteins were identified in GenBank and RefSeq *Clostridium* proteomes using MMSeqs2. *ntnh* gene detection reinforced positive results from SMB or *bont* gene tests in 26 cases (35.62% of the total confirmed cases) of foodborne and infant botulism. In two foodborne cases from the BoNT-diagnostic group, the *ntnh* gene was detected despite negative results from both SMB and *bont* gene tests, highlighting its potential to identify missed cases. An *in silico* analysis of 3,250 RefSeq and 2,494 GenBank annotated *Clostridium* proteomes was conducted. respectively. So, NTNH showed a high co-presence pattern with BoNT. Moreover, NTNH sequences were far more conserved than BoNT sequences in inter-type comparisons (67.2 vs.39.7), which highlights its applicability as a disease biomarker. The *ntnh* gene analysis is a valuable complementary tool enhancing the diagnosis of botulism. The study highlights the need for clear guidelines to interpret positive *ntnh* results when direct toxin or *bont* gene confirmation are negative.

## 1 Introduction

Botulism is a potentially fatal neurotoxin-mediated disease characterized by a symmetrical, descending flaccid paralysis affecting voluntary and autonomic muscles. Many patients develop respiratory compromise, frequently requiring emergent airway support, intensive care, and antitoxin administration. Different forms are distinguished by the route of the toxin entry, such as foodborne botulism (FB), intestinal or infant botulism (IB), as well as wound, inhalational, and iatrogenic (cosmetic or therapeutic) botulisms (Rao et al., 2021). The botulinum neurotoxin (BoNT) is one of the most lethal known substances, produced by seven distinct *Clostridium* species: *Clostridium parabotulinum, Clostridium* sp*orogenes, Clostridium botulinum, Clostridium novyi sensu lato, Clostridium argentinense, Clostridium butyricum*, and *Clostridium baratii*. But, over the latter half of the 20th century, the use of *C. botulinum* to identify any BoNT-producing bacterium gained widespread acceptance (Smith et al., 2023). Based on amino acid variations and antigenic activity, eight toxin types (BoNT/A-BoNT/H) and at least 41 subtypes have been identified in clusters with *ha* or *orf*X conformation (Hill et al., 2007).

Laboratory diagnostic criteria for botulism are based on the isolation of BoNT-producing clostridia, the detection of BoNT, or the identification of their encoding genes (*bont*) https://www.ecdc.europa.eu/en/botulism/facts. A variety of technologies and their derivatives—including cell-based assays, endopeptidase mass spectrometry (Endopep-MS), immunoassays, nucleic acid testing, comparative genomic methods, and electrochemical biosensors—have been developed to detect and characterize the causative agents of botulism (Centurioni et al., 2022; Wang et al., 2025). Diagnosis mainly relies on the mouse bioassay (SMB) and the *bont* gene amplification (Lindström et al., 2001; Pillai et al., 2024). Diagnosis methods possess serious drawbacks. Specifically, SMB requires animal facilities and the *bont* type-specific PCR assays are limited by sequence diversity within subtypes (≥23.7%) (Peck et al., 2017; Pillai et al., 2024). Single nucleotide polymorphisms can affect primer or probe matches, potentially resulting in detection failure (Pillai et al., 2024).

Difficulties in detecting BoNT may be mitigated by identifying the nontoxin-nonhemagglutinin *(ntnh*) gene using a single PCR assay (Raphael and Andreadis, 2007; Fach et al., 2009; Hill et al., 2010; Williamson et al., 2017). All BoNTs are naturally co-expressed with a protective partner, the NTNH protein, forming together the minimal progenitor toxin complex (Lam and Jin, 2015). The role of NTNH in the disease is crucial, as it stabilizes and protects BoNT from the acidic environment and proteases present in the host’s gastrointestinal tract (Gao and Jin, 2024). The *ntnh* gene is highly conserved and is associated with the BoNT complex across all types, subtypes, and variants, but is absent in non-toxigenic strains (Raphael and Andreadis, 2007; Fach et al., 2009; Hill et al., 2010; Williamson et al., 2017). This study evaluates *ntnh* gene detection as a strategy to enhance the molecular diagnosis of botulism.

## 2 Materials and methods

### 2.1 Botulism diagnosis and *ntnh* gene detection

Botulism was diagnosed through SMB and BoNT multiplex PCR on chromosomal extracts obtained from prior enrichment of anaerobic cultures of patient stool samples (Centers for Disease Control and Prevention, 1998; Lindström et al., 2001) at the National Centre of Microbiology (Spain). The *ntnh* gene was studied in suspected botulism cases (Williamson et al., 2017) from a prospective BoNT-diagnostic group (01/2023-08/2025) and retrospectively searched in confirmed botulism cases from a BoNT-historical group (09/2010-12/2022) (Valdezate et al., 2023). This assay also identified the BoNT cluster type (*ha* or *orf*X) (Williamson et al., 2017). Patient characteristics (sex, age, and location) were recorded, stratified by botulism type for both groups.

Clinical samples were collected as part of standard patient care and all data were strictly anonymized prior to analysis, so this study was exempted from formal ethical review and approval by the institutional board. However, all procedures were conducted in accordance with the ethical standards of the Declaration of Helsinki, ensuring the protection of patients’ rights, confidentiality, and the ethical principles relevant to public health investigation. Bioassay was performed in accordance with authorization PROEX (no.252.4/21) following institutional and national animal care guidelines.

### 2.2 *ntnh* gene detection in NCBI genomes

*Clostridium* species proteomes (excluding atypical, metagenome-assembled and multi-isolate project ones), were downloaded from NCBI-Genome repository (https://www.ncbi.nlm.nih.gov/datasets/genome/?taxon=2913503) (Accession: 03/09/2025). Toxin cluster proteins were identified within these proteomes using MMSeqs2 (https://search.mmseqs.com) and representative sequences for toxin subtypes (Lindström et al., 2001) besides for NTNH (GenBank accession number: Q45914), H70 (Q9LBR5.1), HA17 (P46083), HA33 (P0DPR0.1), BotR (WP_011948509.1), P47 (WGZ47456.1), ORFX1 (WGZ47458.1), ORFX2 (WGZ4759.1) and ORFX2 (WGZ4760.1). Identity between homologs was calculated following alignment with Muscle v3.8.1551 (https://www.drive5.com/muscle/). Isolate metadata was downloaded from NCBI Biosample (https://www.ncbi.nlm.nih.gov/biosample). NTNH presence in non-*Clostridium* genomes was explored by BLAST against the RefSeq select database (https://blast.ncbi.nlm.nih.gov/Blast.cgi).

## 3 Results

### 3.1 Study population

Two distinct sample groups were collected: the prospective BoNT-diagnostic group, comprised 88 samples from 77 foodborne botulism (FB) and 11 infant botulism (IB) cases; and the BoNT-historical group, consisting of 54 samples from 35 FB cases and 19 IB cases (**Table 1**).

**Table 1:** Epidemiological characteristics of patients with suspected or confirmed botulism.

Group	Sex	Age range (median)	Location
BoNT^1^-diagnostic group (88)			
FB^2^ (77)	29 females	27–75 yr. (52 yr.)	Andalusia (19), Aragon (1), Asturias (3), Canary Islands (3), Cantabria (1), Catalonia (14), Castile and León (13), Castile-La Mancha (2), Galicia (3), Madrid (12), Melilla (1), Murcia (1), Navarre (1), Valencian Community (8), Basque Country (6)
	48 males	43–75 yr. (55 yr.)	
IB^3^ (11)	6 females	1–12 months (4 months)	
	5 males	3–11 months (5 months)	
BoNT^1^-historical group (54)			
FB^2^ (35)	13 females	14 months-80 yr. (61 yr.)	Andalusia (15), Aragon (3), Baleares (1), Catalonia (13), Castile and León (6), Castile-La Mancha (2), Galicia (1), Madrid (6), Rioja (2), Valencian Community (1), Basque Country (4)
	22 males	19–79 yr. (56 yr.)	
IB^3^ (19)	9 females	20 days-6 months (4 months)	
	10 males	1–9 months (5 months)	

^1^BoNT, botulinum neurotoxin; ^2^FB, foodborne botulism; ^3^IB, infant botulism.

### 3.2 *ntnh* gene detection

In the prospective BoNT-diagnostic group, ten FB cases were *ntnh* positive. Of these, five cases were also positive for SMB and the *bont* gene, while three cases were only *bont* gene positive. The remaining two cases were negative for both the SMB and *bont* tests. Eight cases of IB were *ntnh* positive: four were also positive for SMB and the *bont* gene, one was positive only for the SMB, and three were positive only for the *bont* gene. Regarding the BoNT-historical group, *ntnh* detection was positive in twenty-six FB cases. Of these, eleven cases were positive for both SMB and the *bont* gene, one was positive only for the SMB, and fourteen were positive only for the *bont* gene. Seventeen IB cases were *ntnh* positive: thirteen were positive for both SMB and the *bont* gene, one was positive only for the SMB, and three were positive only for the *bont* gene.

**Figure 1:**
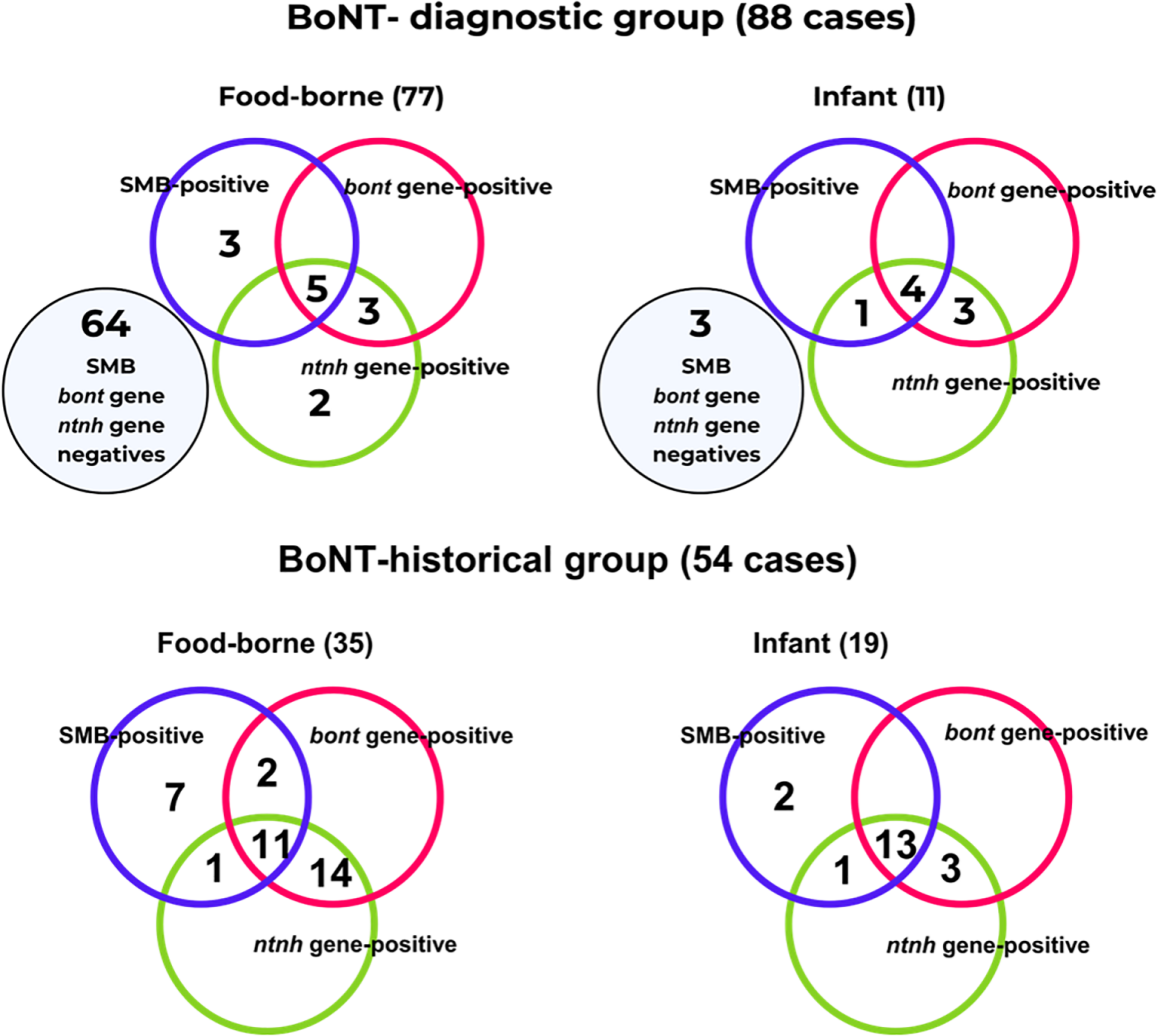
Distribution of *ntnh* detection results across the BoNT-diagnostic group (suspected cases) and the BoNT-historical group (confirmed cases).

Overall, positive *ntnh* gene detection reinforced the positive results obtained by either the SMB or *bont* gene detection in twenty-six cases (35.62%) respect to the positives cases by SMB and/or *bont* genes tests (confirmed botulism). Conversely, the *ntnh* gene failed to detect two FB cases in the historical group, despite positive results from both SMB and *bont* gene tests. Meanwhile, *ntnh* was detected in two FB cases from the BoNT-diagnostic group, that were negative by both the SMB and *bont* gene tests. Data are compiled in **Figure 1** and in **Supplementary Table S2**.

Consistent with the established associations between cluster category and *bont* type (Williamson et al., 2017), our findings showed the following distribution: the *ntnh-ha* cluster was associated with *bont*/A (n=5), *bont*/B (n=45), and *bont*/F (n=1); the *ntnh*-*orf*X cluster was linked to *bont*/A (n=1) and *bont*/F (n=2). In one case of *bont*/A(B), both cluster types were observed.

### 3.3 *ntnh* gene detection in public NCBI genomes

The presence, sequence conservation and co-existence of BoNT and NTNH were assessed using public genomes from updated repositories. Of the 3,250 RefSeq and 2,494 GenBank *Clostridium* genome-based annotated proteomes, 474 and 395, respectively, contained BoNT variants sharing at least 50% identity and 50% alignment length with one BoNT representative. These involved five species previously associated with the BoNT presence. No significant NTNH hits were detected in non-*Clostridium* species.

**Figure 2:**
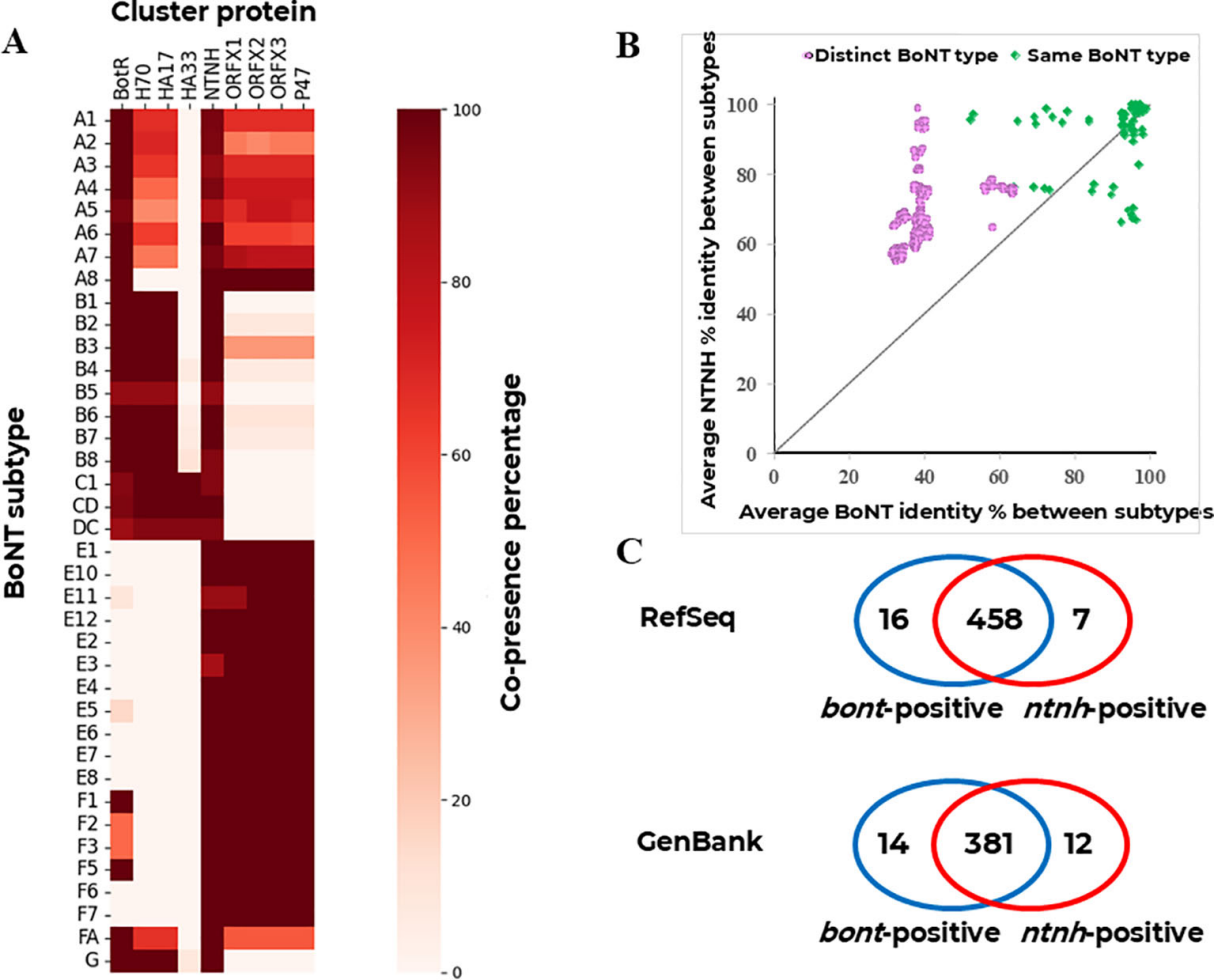
Genome analysis of *bont/ntnh* containing genomes. **(A)** Co-presence of cluster proteins in toxin subtype. **(B)** Inter-subtype sequence identity of *bont* and *ntnh* variants. **(C)** Venn diagrams showing genomic samples with exclusive and overlapping detection of *bont* and *ntnh* genes in RefSeq and GenBank repositories.

Most BoNT cluster proteins showed a strong type association pattern, (i.e. HA proteins with BoNT/A, B, C and D; and OrfX/P47 with BoNT/E, F and G types). The exception was NTNH, which co-existed in 90-100% of strains of all types as appears in color-ranked in the “NTNH” labelled column of **Figure 2A**.

Regarding sequence conservation, NTNH and BoNT homologs showed comparable average identity percentage within BoNT subtypes (90.0 ± 10.6% vs 88.3 ± 11.9%, respectively). In contrast, when homologs from different BoNT subtypes were compared (all-against-all), NTNH was far more conserved than BoNT, particularly when different types were involved (67.2 ± 8.9% vs 39.7 ± 8.4%, respectively) (**Figures 2B, C**). In **Figure 2B**, the majority of the data points—which represent the averaged inter-subtype identities for BoNT and NTNH between toxin subtype pairs—are located above the diagonal of identity. This distribution demonstrates that, in most instances, NTNH sequences exhibit significantly higher conservation than their corresponding BoNT sequences. This trend becomes even more pronounced when comparing subtypes belonging to different toxin types, further supporting our observation that NTNH remains more conserved across divergent lineages. While, **Figure 2C** focuses on the reliability of genomic detection regardless of sequence identity. This approach increases the detection rate of toxigenic strains that might otherwise yield negative results for the *bont* gene.

Furthermore, *ntnh* was detected in seven and twelve BoNT-negative *Clostridium* strains in RefSeq and GenBank genome datasets, respectively. These represented 1.8% and 3.0% increments with respect to BoNT-positive strains and involved only three RefSeq-GenBank duplicated cases. Moreover, metadata of these samples indicated a variety of isolation years, countries, isolation sources and sequencing platforms (**Supplementary Table S1**). Altogether, NTNH detection reinforces the sensitivity of genome-based botulism diagnosis through functional inference.

## 4 Discussion

The effectiveness of molecular tests in reliably detecting all known BoNT subtype variants causing botulism is constrained by the genetic diversity of BoNTs, even within toxin types (Hill et al., 2010; Peck et al., 2017). To address this constraint on accuracy, the detection of NTNH offers an alternative. The *ntnh* gene was exclusively found in toxin-producing clostridia and displayed a high co-presence pattern with *bont* gene. This confirms that the heteromeric stabilized neurotoxin is the actual virulence factor and that *ntnh*-based diagnostic methods are therefore causal. Despite the *ntnh* gene is a known “hotspot” for recombination, insertions, and mosaicism (East et al., 1996; Harris et al., 2024), it was found more conserved than BoNT variants and is thus amenable to widespread detection less multiplexing PCR.

*C. botulinum* has a relatively stable genome except for the *bont* gene cluster where *ntnh* gene is included (Hill et al., 2009). Most of these clusters are flanked by insertion sequence elements encoding transposases that may be capable of horizontal gene transfer between *C. botulinum* Groups I and II, or between species (Smith et al., 2015). Regarding the variability of *ntnh* and *bont* genes across different strains, we observed that NTNH sequences within the same BoNT subtype exhibit a high degree of redundancy. Our results show that within intra-subtype comparisons (representing short-term evolution), BoNT is marginally more conserved than NTNH (99.8 ± 0.3% vs. 97.1 ± 5.7% identity), which is expected as the BoNT variant defines the subtype itself. However, this trend reverses during mid-term evolution (different subtypes within the same type), where NTNH shows higher conservation (90.0 ± 10.6% vs. 88.3 ± 11.9%). This conservation gap is greatly enhanced in long-term evolution (different toxin types), where NTNH identity remains significantly higher than BoNT (67.2 ± 8.9% vs. 39.7 ± 8.4%).

NTNH plays a conserved core role in stabilizing and protecting BoNTs across their diverse natural environments (soil, decaying matter, etc.), forming a stable and large toxin complex (Lam and Jin, 2015) that prevents the toxin from being broken down by enzymes (like proteases) before it can infect a host. However, NTNH is also highly flexible, customizing its structure and function to adapt to either the *ntnh-ha* cluster or the *orfX-ntnh* cluster (a smaller complex). This suggests that NTNH provides an evolutionarily flexible platform for developing new BoNT variants, enabling it to fit into different environments and attack specific host organisms and tissues. The adaptability of NTNH is thus tied directly to the evolutionary progress of BoNTs (Gao and Jin, 2024; Pitel et al., 2025). Consequently, the NTNH protein acts as an “unsung hero” that not only protects the toxin but actively contributes to its evolutionary success and specialization.

While a wide spectrum of PCR assays has been developed to detect *bont* genes, only a limited number of studies have incorporated the *ntnh* gene (Raphael and Andreadis, 2007; Fach et al., 2009; Hill et al., 2010; Williamson et al., 2017). This limited implementation may be due to the fact that *ntnh* detection does not inherently identify the specific toxin serotype. Previous studies have integrated *ntnh* gene into various platforms, including real-time PCR (Raphael and Andreadis, 2007; Fach et al., 2009), quantitative PCR (Hill et al., 2010), and conventional PCR (Williamson et al., 2017). The assay designed by Williamson et al. was selected because it also provides epidemiological information regarding the neurotoxin cluster type and has been validated across *C. botulinum* Group I, *C.* sp*orogenes*, and *C. botulinum* Group II (Williamson et al., 2017).

In botulism diagnostics, discrepancies between the detection of BoNT (the protein) and its corresponding genes (*bont*) are a well-documented event (Centurioni et al., 2022). Here, the application of *ntnh* detection in diagnosis proved to be a useful complementary target for rapidly confirming positive results from the SMB or *bont* PCR (26 cases, 35.62%). High correlation was found between the presence of NTNH and BoNT (96.5% of the analyzed genomes).

A potential limitation of *ntnh* detection is non-expressed toxin genes (Raphael and Andreadis, 2007), although this event does not occur in our study, with the detection of *ntnh-ha* and *ntnh-orf*X clusters in a BoNT/A1(B5) case. Furthermore, the *ntnh* assay provides only indirect evidence for BoNT presence and relies on the functional assumption that BoNT and NTNH are mutually required. While the current criteria for laboratory confirmation of botulism requires explicit positive results from the SMB and/or *bont* gene identification, there are no established guidelines for interpreting a positive *ntnh* gene detection when both the SMB and *bont* tests are negative. This gap in guidelines is highlighted by its occurrence in two patients within BoNT- diagnostic group who were later clinically diagnosed with food-borne botulism.

In conclusion, the detection of *ntnh* represents a valuable addition to our diagnostic arsenal of botulism. Our findings suggest the need for establishing guidelines on how to interpret a positive *ntnh* result in the absence of direct toxin and *bont* gene confirmation to maximize the potential of this target.

## Data Availability

The datasets presented in this study can be found in online repositories. The names of the repository/repositories and accession number(s) can be found in the article/**Supplementary Material**.
