# Supplementary material for: The unsung hero: ntnh gene as complementary botulism marker
Source: Front Cell Infect Microbiol. 2026 Feb 23;16:1758429. doi: 10.3389/fcimb.2026.1758429 (PMC12968224; doi:10.3389/fcimb.2026.1758429)
Supplement: Supplementary file 1 [file Table1.docx]

Supplementary Material

# Supplementary Table 1. The *ntnh* gene detection and its correlation with botulism diagnosis cases (n=142).

| Group and botulism type (no. of cases) | SMB-*bont*-*ntnh* results | No. of cases | *bont* gene type or subtype (no. of cases) | *ntnh* gene type |
| --- | --- | --- | --- | --- |
| BoNT-diagnostic group (88) | |  |  |  |
| Food-borne (77) | SMB (+) *bont* (+) *ntnh* (+) | 5 | *bont*/A (1)  *bont*/B2 (3) B (1) | *ntnh*-*orfX  ntnh*-ha |
|  | SMB (+) *bont* (-) *ntnh* (-) | 3 | no detected | no detected |
|  | SMB (-) *bont* (+) *ntnh* (+)^1^ | 3 | *bont*/A (1) *bont*/B2 (1)  *bont*/A1(B5) (1) | *ntnh*-ha  *ntnh*-ha  *ntnh*-ha/*ntnh*-orfX |
|  | SMB (-) *bont* (-) *ntnh* (+)^2^ | 2 | no detected | *ntnh*-ha |
|  | SMB (-) *bont* (-) *ntnh* (-) | 64 | no detected | no detected |
| Infant (11) | SMB (+) *bont* (+) *ntnh* (+) | 4 | *bont*/B2(3), B(1) | *ntnh*-ha |
|  | SMB (+) *bont* (-) *ntnh* (+)^1^ | 1 | no detected | *ntnh*-*orfX* |
|  | SMB (-) *bont* (+) *ntnh* (+)^1^ | 3 | *bont*/B2(2), B(1) | *ntnh*-ha |
|  | SMB (-) *bont* (-) *ntnh* (-) | 3 | no detected | no detected |
| BoNT-historical group (54 cases) | |  |  |  |
| Food-borne (35) | SMB (+) *bont* (+) *ntnh* (+) | 11 | *bont* /A1 (1), A2 (1)  *bont* /B2 (6), B(1)  *bont* /F7 (2) | *ntnh-*ha  *ntnh-*ha  *ntnh-orfX* |
|  | SMB (+) *bont* (+) *ntnh* (-)^3^ | 2 | *bont* /B (2) | no detected |
|  | SMB (+) *bont* (-) *ntnh* (+)^1^ | 1 | no detected | *ntnh-*ha |
|  | SMB (+) *bont* (-) *ntnh* (-) | 7 | no detected | no detected |
|  | SMB (-) *bont* (+) *ntnh* (+)^1^ | 14 | *bont* /A (2) *bont* /B2 (10) B(1) *bont* /F8 (1) | *ntnh-*ha  *ntnh-*ha  *ntnh-orfX* |
| Infant (19) | SMB (+) *bont* (+) *ntnh* (+) | 13 | *bont* /B2 (13) | *ntnh-*ha |
|  | SMB (+) *bont* (-) *ntnh* (+)^1^ | 1 | no detected | *ntnh-orfX* |
|  | SMB (+) *bont* (-) *ntnh* (-) | 2 | no detected | no detected |
|  | SMB (-) *bont* (+) *ntnh* (+)^1^ | 3 | *bont* /B (3) | *ntnh-*ha |

^1^the *ntnh* gene detection reinforced the positive results obtained by either the SMB or *bont* gene; ^2^the *ntnh* gene was detected, although both the SMB and *bont* gene tests were negative; ^3^the *ntnh* gene fails in the detection, despite positive results from both SMB and *bont* gene tests.

# Supplementary Table 2. Characteristics of NTNH-positive and BoNT*-*negative GenBank and RefSeq genomes identified in this study.

| Assembly | Database | Biosample | Genome submission | Sequencing platform | Year of isolation | Country | Source |
| --- | --- | --- | --- | --- | --- | --- | --- |
| GCA_16071555.1 | GenBank | SAMN16288619 | 19/11/2020 | PacBio RSII | 1958 | Chad | Ham |
| GCA_17330695.1 | GenBank | SAMN10922119 | 14/02/2019 | IonTorrent | 1976 | USA:CA | Stool |
| GCA_17330965.1 | GenBank | SAMN10922105 | 14/02/2019 | IonTorrent | 1982 | USA:Hawaii | Stool |
| GCA_17331025.1 | GenBank | SAMN10922101 | 14/02/2019 | IonTorrent | 1983 | USA:Hawaii | Wound |
| GCA_17330785.1 | GenBank | SAMN10922111 | 14/02/2019 | IonTorrent | 1992 | USA:Hawaii | Stool |
| GCA_17330795.1 | GenBank | SAMN10922113 | 14/02/2019 | IonTorrent | 2008 | USA:Hawaii | Stool |
| GCA_307655.1 | GenBank | SAMN01816399 | 07/12/2012 | IonTorrent | 2008 | USA:Miami | Water |
| GCA_17330715.1 | GenBank | SAMN10922118 | 14/02/2019 | IonTorrent | 2010 | USA:Hawaii | Stool |
| GCA_17330665.1 | GenBank | SAMN10922117 | 14/02/2019 | IonTorrent | 2015 | USA:Hawaii | Stool |
| GCF_17330945.1 | GenBank/RefSeq | SAMN10922106 | 14/02/2019 / 06/11/2024 | IonTorrent | 1983 | USA:Hawaii | Stool |
| GCF_3014955.1 | GenBank/RefSeq | SAMN08619546 | 20/03/2018 / 12/04/2025 | Illumina MiSeq | 2015 | China: Hebei | Infant rice cereal |
| GCF_3058445.1 | GenBank/RefSeq | SAMN07135561 | 13/04/2018 / 12/04/2025 | PacBio | 2017 | USA:IL | NA |
| GCF_730785.1 | RefSeq | SAMN02664949 | 13/04/2025 | IonTorrent | 1993 | USA:Georgia | Swab |
| GCF_1266885.1 | RefSeq | SAMN03876600 | 06/06/2025 | Illumina MiSeq | 2008 | France:Loir et Cher | Chicken |
| GCF_20819655.1 | RefSeq | SAMN22814977 | 30/08/2025 | Illumina MiSeq | 2016 | Russia | Canned cucumber |
| GCF_353835.1 | RefSeq | SAMN02471792 | 06/06/2025 | 454 GS 20; 454 GS FLX | No available | Finland | Finish food, fish roe |
